# Spiculisporic Acids B–D, Three New *γ*-Butenolide Derivatives from a Sea Urchin-Derived Fungus *Aspergillus* sp. HDf2

**DOI:** 10.3390/molecules171113175

**Published:** 2012-11-05

**Authors:** Rong Wang, Tian-Mi Liu, Ming-Hui Shen, Ming-Qiu Yang, Quan-Ying Feng, Xian-Ming Tang, Xiang-Min Li

**Affiliations:** Hainan Provincial Fisheries Research Institute, Haikou 570203, Hainan, China

**Keywords:** *γ*-butenolide derivatives, spiculisporic acids B–D, *Aspergillus* sp., marine fungus

## Abstract

Three new *γ*-butenolide derivatives **1**–**3**, named spiculisporic acids B–D, were isolated from the culture of *Aspergillus* sp. HDf2, a marine-derived fungus that resides in the sea urchin, *Anthocidaris crassispina*. The structures of **1**–**3** were elucidated on the basis of spectroscopic methods, including MS and 2D NMR techniques. Their *in vitro* cytotoxic activities against two cell lines (SGC-7901, human gastric adenocarcinoma and SPC-A-1, human lung adenocarcinoma) and inhibitory activities against *Staphylococcus aureus* ATCC 51650 were investigated.

## 1. Introduction

**Figure 1:**
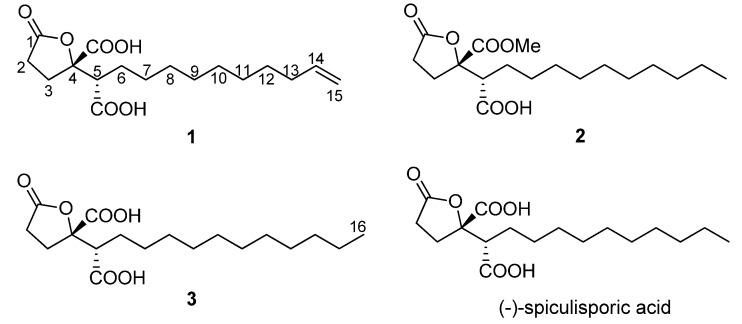
Structures of compounds **1**–**3**.

Microorganisms of marine origin have proven to be a rich source of novel and/or biologically active natural products with promising pharmacological properties [1,2,3,4]. In related studies, the Tsukamoto laboratory reported a new antimicrobial anthraquinone (monodictyquinone A) from a sea urchin-derived fungus *Monodictys* sp. [5]. In our search for new natural products from marine-derived microorganisms, the fungus *Aspergillus* sp. HDf2 was isolated from the sea urchin *Anthocidaris crassispina*, collected from the seashore of Qionghai, Hainan, China. The genus *Aspergillus* (Trichocomaceae) is one of the most prolific fungi that produce a variety of secondary metabolites with novel structures and interesting bioactivities [6]. Subsequent chemical study on the fermentation broth of the fungus *Aspergillus* sp. HDf2 led to the isolation of three new *γ*-butenolide derivatives, the structures of which were similar to that of spiculisporic acid [7,8], and these compounds were thus named as spiculisporic acids B–D (compounds **1**–**3**, Figure 1). Their structures were elucidated by comprehensive spectroscopic analyses. Herein we report the isolation, structural determination, bioactivities of these new natural products.

## 2. Results and Discussion

**Figure 2:**
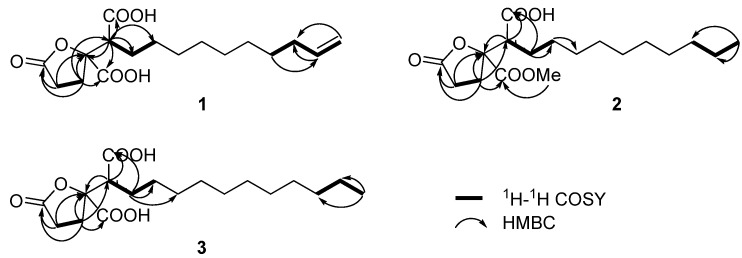
^1^H-^1^H COSY and selected HMBC correlations of compounds **1**–**3**.

Spiculisporic acid B (**1**) was isolated as a white solid, with the molecular formula C_17_H_26_O_6_ (five degrees of unsaturation) as derived from ESI high-resolution mass spectrometry ([M−H]^−^ at *m/z* 325.1664, calculated 325.1657) and ^1^H- and ^13^C-NMR spectral data (Table 1 and Table 2). The ^13^C-NMR showed three carbonyl carbons at *δ*_C_ 178.5, 175.5, and 174.2, one olefinic methine carbon at *δ*_C_ 140.2, one olefinic methylene carbon at *δ*_C_ 114.8, one oxygen bearing quaternary carbon at *δ*_C_ 88.2, one methine carbon at *δ*_C_ 52.5, and ten aliphatic carbons in the upfield (*δ*_C_ 35.0 to 28.9) region. The ^1^H-NMR spectrum displayed signals of one terminal vinyl group at *δ*_H_ 5.81 (ddt, *J* = 16.1, 10.2, 6.8 Hz, H-14), 4.98 (br d, *J* = 16.1 Hz, H-15a), and 4.91 (br d, *J* = 10.2 Hz, H-15b), and 21 aliphatic protons. Together, these data indicate that compound **1** has one double bond and three carbonyls, which account for 4 out of the 5 degrees of unsaturation required by the molecular formula, so spiculisporic acid B must contain a ring. The structural information for **1** was determined from a series of 2D NMR analyses, including HSQC, ^1^H-^1^H COSY, and HMBC spectra (Figure 2). The ^1^H-^1^H COSY experiment revealed a correlation between H-2 (*δ*_H_ 2.60) and H-3 (*δ*_H_ 2.49), and a seperate spin system, H_2_C=CH-CH_2_-(CH_2_)_6_-CH_2_-CH-. The methine proton H-14 was coupled with the methylene protons H-13 (*δ*_H_ 2.04) and H-15, and the methylene proton H-13 was coupled with the methylene proton at H-12 (*δ*_H_ 1.37). The correlations between the methylene proton H-6a (*δ*_H_ 1.85) and H-5 [*δ*_H_ 3.01 (br d, *J* = 9.2 Hz)] and H-7 (*δ*_H_ 1.25–1.37) were observed in the ^1^H-^1^H COSY spectrum. HMBC correlations from H-5 to C-4 (*δ*_C_ 88.2) and two carbonyl carbons (*δ*_C_ 175.5 and 174.2), from H-3 to C-1 (*δ*_C_ 178.5), C-4, C-5 (*δ*_C_ 52.5), and one of the carbonyl carbons (*δ*_C_ 174.2), and from H-2 to C-1 and C-4, were observed. These observations allowed the structure of **1** to be determined as shown in Figure 1. 

**Table 1:** ^1^H-NMR spectral data (500 MHz, CD_3_OD) of compounds **1**–**3**.

Position	1	2	3
2	2.60 (m)	2.59 (m)	2.59 (m)
3	2.49 (m)	2.48 (m)	2.46 (m)
4-COOMe		3.80 (s)	
5	3.01 (br d, *J* = 9.2)	2.97 (dd, *J* = 10.8, 2.5)	3.03 (dd, *J* = 11.0, 3.0)
6	1.85 (m); 1.50 (m)	1.82 (m); 1.51 (m)	1.85 (m); 1.53 (m)
7	1.25–1.37 (m) *^a^*	1.43 (m); 1.32 (m)	1.42 (m); 1.32 (m)
8	1.25–1.37 (m) *^a^*	1.25–1.37 (m) *^b^*	1.25–1.38 (m) *^c^*
9	1.25–1.37 (m) *^a^*	1.25–1.37 (m) *^b^*	1.25–1.38 (m) *^c^*
10	1.25–1.37 (m) *^a^*	1.25–1.37 (m) *^b^*	1.25–1.38 (m) *^c^*
11	1.25–1.37 (m) *^a^*	1.25–1.37 (m) *^b^*	1.25–1.38 (m) *^c^*
12	1.37 (m)	1.25–1.37 (m) *^b^*	1.25–1.38 (m) *^c^*
13	2.04 (m)	1.25–1.37 (m) *^b^*	1.25–1.38 (m) *^c^*
14	5.81 (ddt, *J* = 16.1, 10.2, 6.8)	1.25–1.37 (m) *^b^*	1.25–1.38 (m) *^c^*
15	4.98 (br d, *J* = 16.1) 4.91 (br d, *J* = 10.2)	0.90 (t, *J* = 6.8)	1.25–1.38 (m) *^c^*
16			0.90 (t, *J* = 7.0)

*^a^^−c^* Overlapping signals.

**Table 2:** ^13^C-NMR spectral data (125 MHz, CD_3_OD) of compounds **1**–**3**.

Position	1	2	3
1	178.5 (s)	178.0 (s)	178.7 (s)
2	28.9 (t) *^a^*	28.7 (t)	29.0 (t) *^e^*
3	30.3 (t)	30.5 (t)	30.6 (t) *^d^*
4	88.2 (s)	88.2 (s)	88.7 (s)
4-COOH	174.2 (s)		174.9 (s)
4-CO		172.8 (s)	
OMe		53.6 (q)	
5	52.5 (d)	52.9 (d)	52.7 (d)
5-COOH	175.5 (s)	175.2 (s)	175.8 (s)
6	29.2 (t)	28.9 (t) *^b^*	29.1 (t)
7	28.9 (t) *^a^*	28.9 (t) *^b^*	29.0 (t) *^e^*
8	30.7 (t) *^f^*	30.5 (t) *^c,g^*	30.6 (t) *^ d^*
9	30.4 (t) *^f^*	30.4 (t) *^g^*	30.6 (t) *^d,h^*
10	30.5 (t) *^f^*	30.5 (t) *^c,g^*	30.8 (t) *^h^*
11	30.5 (t) *^f^*	30.8 (t) *^g^*	30.9 (t) *^h^*
12	30.2 (t)	30.7 (t) *^g^*	30.7 (t) *^h^*
13	35.0 (t)	33.1 (t)	30.5 (t)
14	140.2 (d)	23.8 (t)	33.2 (t)
15	114.8 (t)	14.5 (q)	23.8 (t)
16			14.5 (q)

*^a^^−e^* Overlapping signals. *^f^^−h^* Interchangeable signals.

To confirm the absolute configuration of **1**, through comparison of its chemical shifts for C-4 (*δ*_C_ 88.2) and C-5 (*δ*_C_ 52.5) with those of the known compounds (−)-spiculisporic acid (*δ*_C_ 88.1, 52.5) and (−)-epispiculisporic acid (*δ*_C_ 88.0, 51.9) [7,8], we tentatively propose the absolute configuration at C-4 and C-5 in **1** to be 4*S* and 5*S* as (−)-spiculisporic acid. Thus, the structure was identified as (4*S*,5*S*)-4-(5-carboxyl-undecyl-14-enyl)-1-oxo-tetrahydrofuran-4-carboxyl acid, named spiculisporic acid B. 

Spiculisporic acid C (**2**) was obtained as a waxy solid that analyzed for the molecular formula C_18_H_30_O_6_ by HR-ESI-MS data ([M+Na]^+^* m/z* 365.1927), and by comprehensive analysis of NMR data. This formula differed by the addition of CH_2_ to the molecular formula of spiculisporic acid [7,8], suggesting an additional methylene or methyl group had been added to the structure. The ^1^H and ^13^C-NMR data for **2** were almost identical to those of spiculisporic acid, except for the presence of a new oxygenated methyl group (*δ*_C_ 53.6, *δ*_H_ 3.80). The HMBC spectrum showed a strong correlation between the oxygenated methyl protons and carbonyl carbon at *δ*_C_ 172.8, which was correlated with H-3 (*δ*_H_ 2.48), thus indicating the position of the methoxyl group. Based on the HSQC, ^1^H-^1^H COSY, and HMBC analyses of **2 **(Figure 2), and the good comparison of NMR data of C-4 (*δ*_C_ 88.2) and C-5 (*δ*_C_ 52.9) in **2** to those from (−)-spiculisporic acid, we proposed the structure of **2** to be (4*S*,5*S*)-4-(5-carboxyl-undecyl)-1-oxo-tetrahydrofuran-4-carboxyl acid methyl ester, named spiculisporic acid C. It was possible that the methoxyl group in **2** was a result of a reaction with methanol in the procedure of isolation. 

Spiculisporic acid D (**3**), isolated as a pale white solid, gave a [M+Na]^+^ ion peak at *m/z* 365.1940 in its positive-mode HR-ESI-MS, indicating its molecular formula to be C_18_H_30_O_6_, which was the same as that of **2**. Through detailed analyses of the ^1^H- and ^13^C-NMR spectra of **3**, the major difference between **3** and spiculisporic acid was the presence of a new methylene group in the aliphatic chain. Unambiguous assignments of ^1^H- and ^13^C-NMR data were obtained by interpretation of HSQC, ^1^H-^1^H COSY, and HMBC data (Figure 2), confirming the structure for **3** as shown. The absolute configuration of **3**, determined by comparison of NMR data of C-4 (*δ*_C_ 88.7) and C-5 (*δ*_C_ 52.7) in **3**with those of (−)-spiculisporic acid, was elucidated as (4*S*,5*S*)-4-(5-carboxyl-dodecyl)-1-oxo-tetrahydrofuran-4-carboxyl acid, named spiculisporic acid D. 

Compounds **1**–**3** were subjected to cytotoxic activity tests against two cell lines, SGC-7901 and SPC-A-1 by MTT methods [9]. However, none of these compounds were active with IC_50_ > 50 μg/mL. Compounds **1**–**3** showed antibacterial activities against *Staphylococcus aureus* ATCC 51650 with inhibition zone of 9.6, 11.6, and 11.5 mm at 20 mg/mL, while the diameter of inhibition zone of the positive control was 23.6 mm. Spiculisporic acid, a fermentation adduct from the culture broth of *Penicillium spiculisporum* has found potential use as new controlled release carriers of active chemicals [7], and commercial application as a biosurfactant for metal decontamination processes to remove hard, large metal cations from water [10]. These interesting properties of spiculisporic acids B–D are currently under investigation.

## 3. Experimental

### 3.1. General Experimental Procedures

Optical rotations were taken on a Rudolph Autopol III. UV spectra were measured on a Hitachi U-3000 spectrophotometer, and IR spectra (KBr) were obtained on a Nicolet 380 FT-IR spectrometer. NMR spectra were recorded on a Bruker AVIII-500 spectrometer at 500 MHz for ^1^H-NMR and at 125 MHz for ^13^C-NMR. Chemical shifts are given in *δ* (ppm) and referenced to the solvent signal (methanol-*d*_4_, *δ*_H_ 3.31, *δ*_C_ 49.1) as the internal standard, and coupling constants (*J*) are reported in Hz. HR-ESI-MS spectra were recorded on a Agilent 6210 TOF LC/MS mass spectrometer. Silica gel (200–300 mesh) for column chromatography (CC) and silica GF_254_ (10–20 mm) for TLC were obtained from Qingdao Marine Chemical Factory (Qingdao, China). YMC ODS gel (50 μm) was purchased from Shanghai HANKING Instrument & Equipment Co., Ltd. (Shanghai, China) Sephadex LH-20 for chromatography was purchased from Merck (Darmstadt, Germany). Semipreparative HPLC was performed on a Hitachi L-7110 pump, and UV detector L-7400 equipped with a Waters ODS column (5 μm, 250 × 4.6 mm).

### 3.2. Fungal Material and Cultivation

The fungus *Aspergillus* sp. HDf2 was isolated and identified by one of the authors (R.W.) from the gut of a healthy sea urchin *Anthocidaris crassispina* collected from the seashore of Qionghai, Hainan, China, in October 2009. A voucher specimen with the code HNF-HD02 is deposited in the Hainan Provincial Fisheries Research Institute. The fungus was cultivated on MEA solid medium composed of 20 g/L malt extract, 20 g/L sucrose, 1 g/L peptone, 20 g/L agar and deionized water for 5 days at 28 °C. Agar plugs were used to inoculate in 1000-mL Erlenmeyer flasks, each containing 300 mL of ME liquid media. Fermentation was carried out on a rotary shaker (140 rpm) at 26 °C for 12 days in 40 × 1,000 mL Erlenmeyer flasks.

### 3.3. Extraction and Isolation

The filtrate (12 L) of the fermented culture broth was extracted three times with EtOAc (12 L × 4) at room temperature, and the organic solvent was evaporated to dryness under reduced pressure to afford a yellow crude extract (4.1 g), which was subjected to silica gel (41 g, 200–300 mesh) CC (4 × 75 cm) eluted with a gradient of CHCl_3_–MeOH (v/v 100:0, 100:1, 100:2, 100:4, 100:8, 100:16 and 0:100, each 600 mL) to give seven fractions. The CHCl_3_–MeOH (100:4) fraction (710.3 mg) was further purified by Sephadex LH-20 CC (1.5 × 30 cm) eluting with MeOH (500 mL) and then by ODS CC (2.5 × 40 cm) with a gradient of MeOH-H_2_O (v/v 50:50, 65:35, 80:20, 100:0, each 400 mL) to afford a fraction (110.5 mg) (MeOH–H_2_O, 80:20) containing **1**–**3**, which were purified by semipreparative reversed-phase HPLC [2 mL/min; MeOH-0.1% TFA in H2O (78:22)] (**1**, 10.3 mg, *t*_R_ = 16.0 min; **2**, 16.2 mg, *t*_R_ = 27.8 min; **3**, 22.6 mg, *t*_R_ = 29.6 min). All these compounds were stored at 4 °C.

*Spiculisporic acid* *B* (**1**): (4*S*,5*S*)-4-(5-Carboxylundecyl-14-enyl)-1-oxo-tetrahydrofuran-4-carboxylic acid. White solid; [*α*]^30^_D_ = −4.8 (c = 0.028, EtOH); UV (MeOH) *λ*_max_ (log *ε*): 199 (3.07), 215 (3.89) nm; IR (KBr) *ν*_max_: 2912, 2853, 1711, 1688, 1415, 1272, 1175, 932 cm^−1^; ^1^H and ^13^C-NMR spectral data are listed in Table 1 and Table 2; HR-ESI-MS: *m/z* 325.1664 [M−H]^−^ (calculated for C_17_H_25_O_6_, 325.1657).

*Spiculisporic acid* *C* (**2**): (4*S*,5*S*)-4-(5-Carboxylundecyl)-1-oxo-tetrahydrofuran-4-carboxylic acid methyl ester. Waxy solid; [*α*]^30^_D_ = −24.7 (c = 0.078, EtOH); UV (MeOH) *λ*_max_ (log *ε*): 214 (3.18) nm; IR (KBr) *ν*_max_: 2921, 2855, 1716, 1663, 1412, 1274, 1183, 952 cm^−1^; ^1^H and ^13^C-NMR spectral data are listed in Table 1 and Table 2; HR-ESI-MS: *m/z* 365.1927 [M+Na]^+^ (calculated for C_18_H_30_O_6_Na, 365.1935).

*Spiculisporic acid* *D* (**3**): (4*S*,5*S*)-4-(5-Carboxyldodecyl)-1-oxo-tetrahydrofuran-4-carboxylic acid. Pale white solid; [*α*]^30^_D_ = −11.8 (c = 0.028, EtOH); UV (MeOH) *λ*_max_ (log *ε*): 212 (3.22) nm; IR (KBr) *ν*_max_: 2918, 2860, 1721, 1657, 1423, 1268, 1175, 944 cm^−1^; ^1^H and ^13^C-NMR spectral data are listed in Table 1 and Table 2; HR-ESI-MS: *m/z* 365.1940 [M+Na]^+^ (calculated for C_18_H_30_O_6_Na, 365.1935).

### 3.4. *In Vitro* Cytotoxicity Test

The cytotoxic activities for compounds **1**–**3** were tested *in vitro* against two cell lines, SGC-7901 (human gastric adenocarcinoma) and SPC-A-1 (human lung adenocarcinoma), which were purchased from the Jiangsu Provincial Center for Disease Prevention and Control. The purity of the tested compounds and doxorubicin·HCl was determined to be over 95% by using the HPLC-DAD method. The cytotoxic *in vitro* effects on these tested cell were assessed by the IC_50_ values, and determined by the MTT [3-(4,5-dimethylthiazol-2-yl)-2,5-diphenyltetrazolium bromide] colometric method [8]. Each set of test was conducted three times to confirm reproducibility of the results. The compounds were dissolved in DMSO (dimethyl sulfoxide). Doxorubicin·HCl was used as a positive control, and the medium without test compound as a negative control in the bioassay.

### 3.5. Antibacterial Test

Compounds **1**–**3** were tested for *in vitro* antimicrobial activity against *Staphylococcus aureus* ATCC51650 by the filter paper disc agar diffusion method. The NA medium was mixed with 2 mL of suspension containing 1 × 10^5^ ~ 1 × 10^7^ cfu/mL of *Staphylococcus aureus*, and then poured into petri-plates. 2 μL 20 mg/mL of the isolated compounds dissolved in DMSO were impregnated on sterile filter paper discs (6 mm diameter) and then were applied on the surface of the solidified agar plates. Every sample was tested in triplicate. Streptomycin sulfate (2 μL, 20 mg/mL) was used as positive control. The test plates were incubated at 37 °C for 24 h. Then the diameters of the inhibition zones including the 6 mm disc diameter were measured.

## 4. Conclusions

In our screening for new secondary metabolites from marine-derived fungi associated with marine animals from the coast of Hainan Island, three new secondary metabolites **1**–**3**, named spiculisporic acids B–D were isolated from a sea urchin (*Anthocidaris crassispina*)-associated fungus *Aspergillus* sp. HDf2 for the first time and characterized. Their structures were elucidated by NMR spectroscopic methods, and the absolute configurations were determined by comparing the chemical shifts of their chiral carbons with those of related known analogues. The compounds displayed no cytotoxic activity against human gastric adenocarcinoma cell line SGC-7901 and human lung adenocarcinoma cell line SPC-A-1 with IC_50_ > 50 μg/mL. Compounds **1**–**3** showed weak antibacterial activities against *Staphylococcus aureus* ATCC 51650 at 20 mg/mL. Further research into their new activities is in progress.

## Supplementary Materials

Supplementary materials can be accessed at: http://www.mdpi.com/1420-3049/17/11/13175/s1.
