# Supplementary material for: Spiculisporic Acids B–D, Three New γ-Butenolide Derivatives from a Sea Urchin-Derived Fungus Aspergillus sp. HDf2
Source: Molecules. 2012 Nov 5;17(11):13175–82. doi: 10.3390/molecules171113175 (PMC6268229; doi:10.3390/molecules171113175)
Supplement: Supplementary file 1 [file molecules-17-13175-s001.pdf]

Supporting Information

NMR spectra of compounds 1–3

Figure S1. The ^{1}H-NMR spectrum of compound **1** (methanol-d_4, 500 MHz).

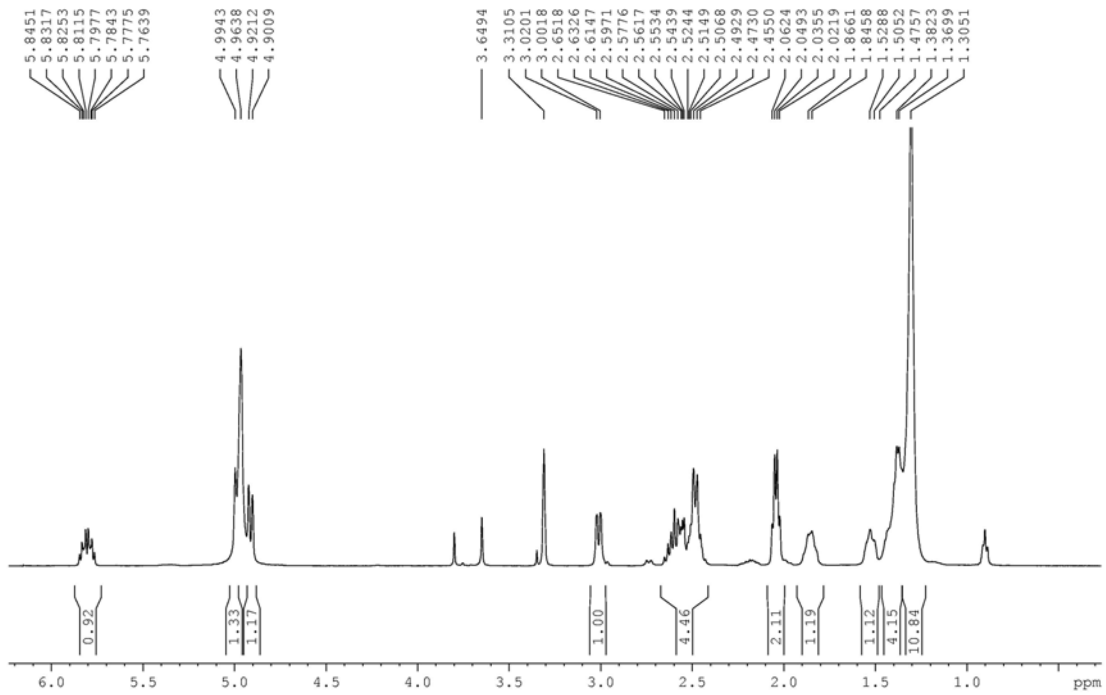

Figure S2. The ^{13}C-NMR spectrum of compound **1** (methanol-d_4, 125 MHz).

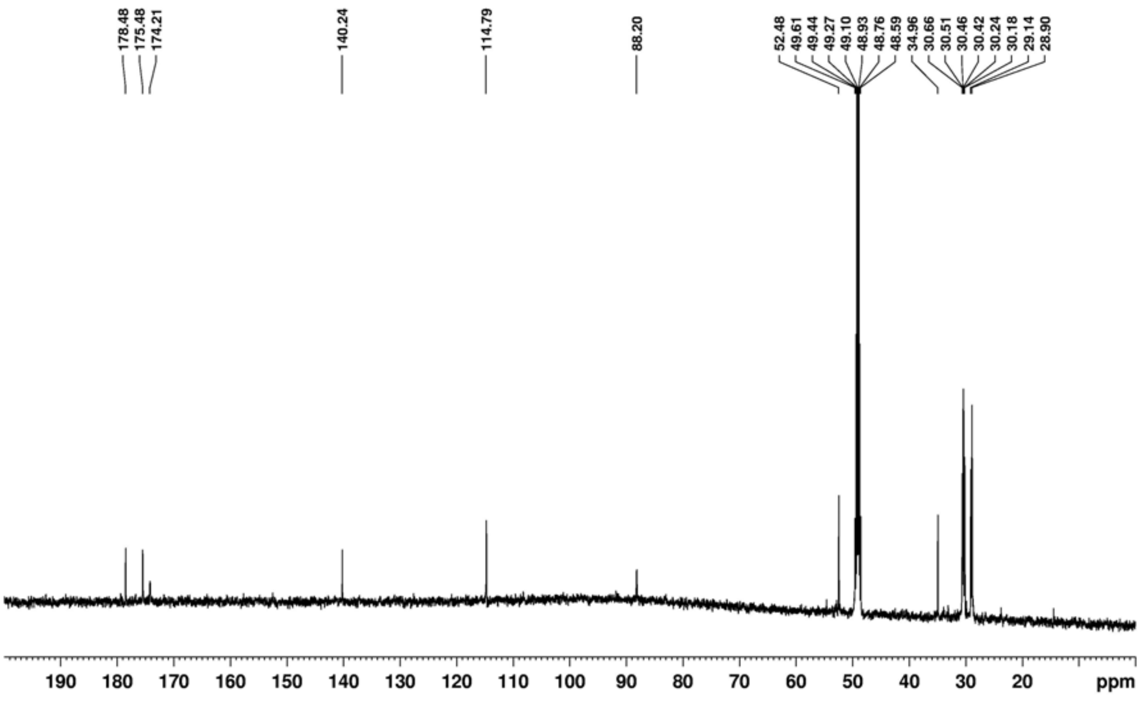

Figure S3. The DEPT135 NMR spectrum of compound **1** (methanol-d_4, 125 MHz).

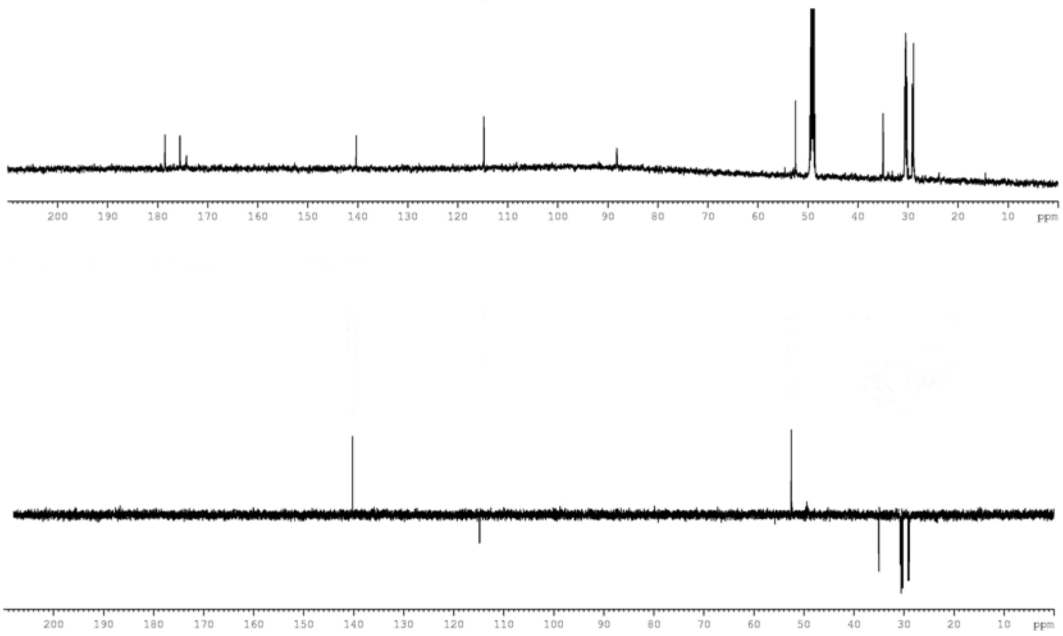

Figure S4 The HRESIMS spectrum of compound **1.**

Qualitative Analysis Report

**Data Filename**	HaDaf2-D1-7-2-2N.d	**Sample Name**	HaDaf2-D1-7-2-2
**Sample Type**	Sample	**Position**	P1-A5
**Instrument Name**	Instrument 1	**User Name**	
**Acq Method**	TEMP2.m	**Acquired Time**	2/25/2011 3:15:29 PM
**IRM Calibration Status**	Some Ions Missed	**DA Method**	Default.m
**Comment**			

User Spectra

Fragmentor Voltage	Collision Energy	Ionization Mode
170	0	Esi

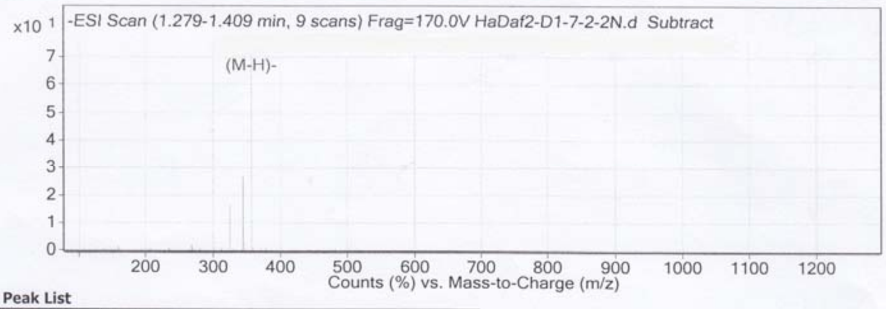

Peak List

m/z	z	Abund	Formula	Ion
325.1664		104109		
345.1917		171723		
357.1918	1	473118		
358.1956	1	73902		

--- End Of Report ---

Figure S5. The ^{1}H-NMR spectrum of compound **2** (methanol-d_4, 500 MHz).

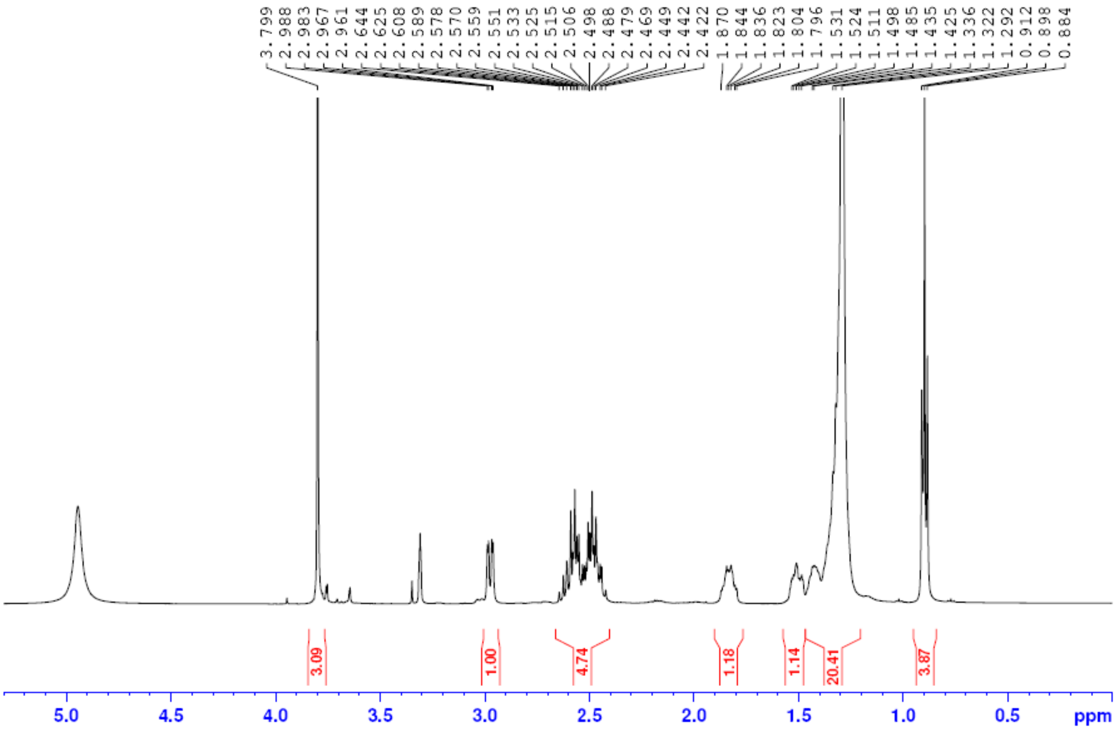

Figure S6. The ^{13}C-NMR spectrum of compound **2** (methanol-d_4, 125 MHz).

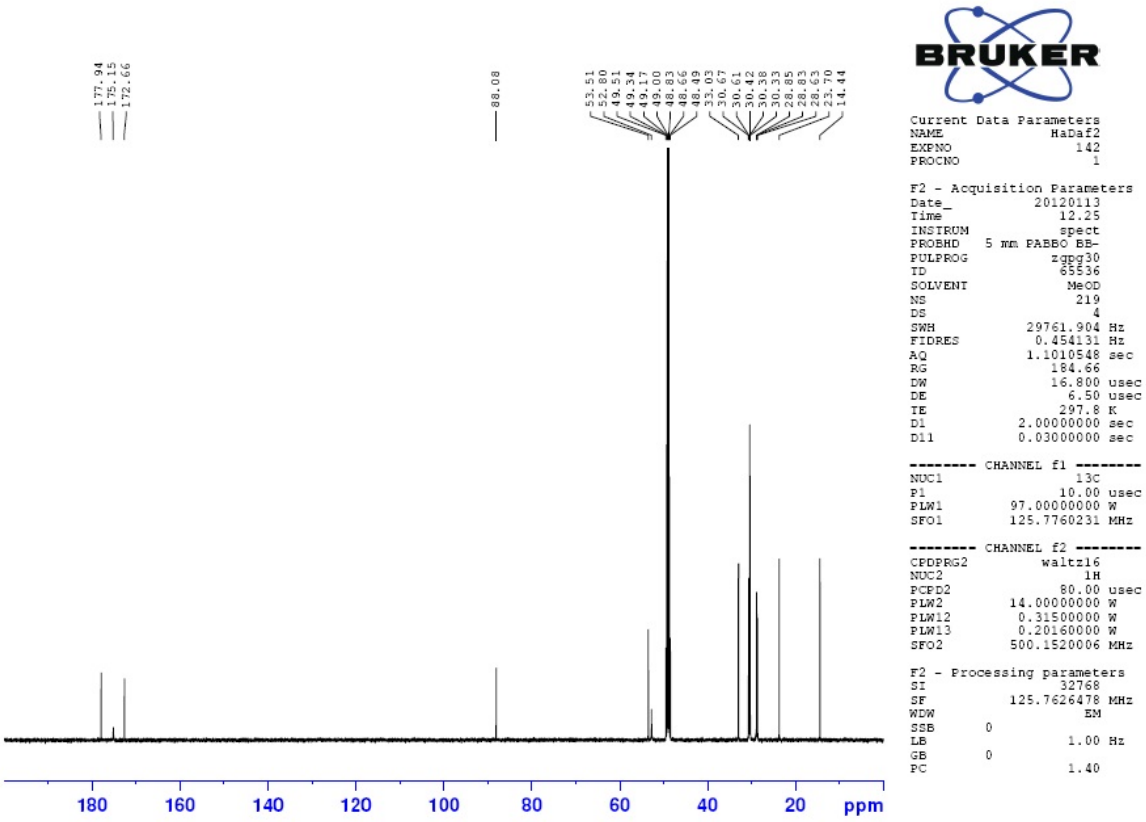

Figure S7. The DEPT135 NMR spectrum of compound **2** (methanol-d_4, 125 MHz).

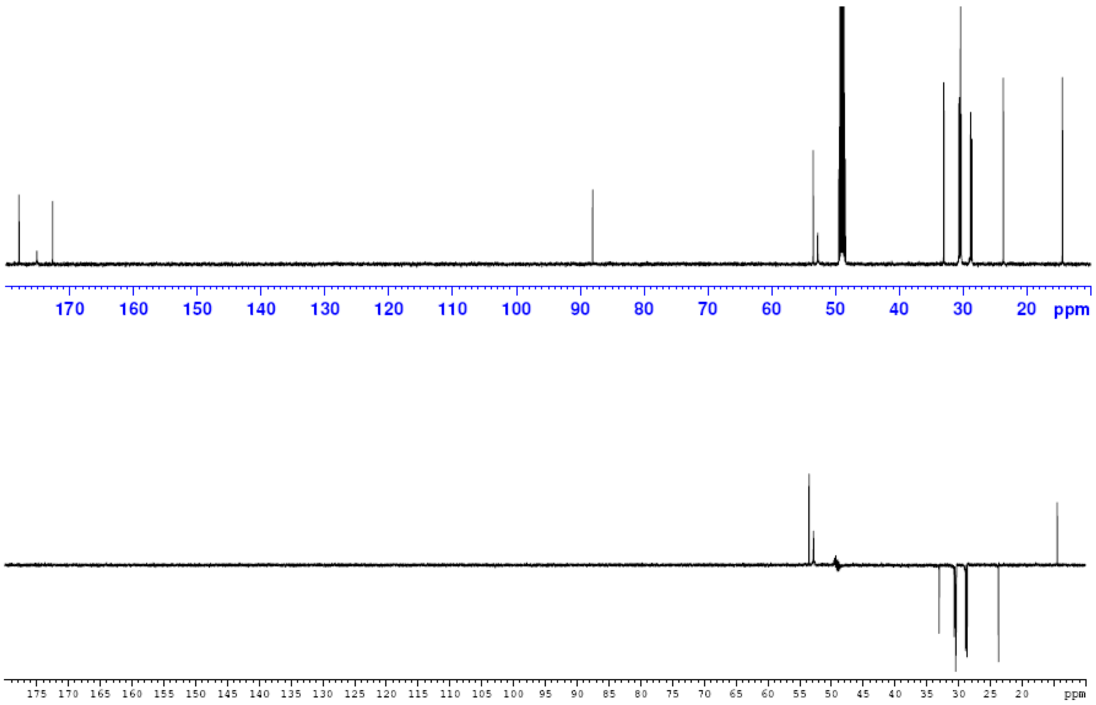

Figure S8. The HRESIMS spectrum of compound **2.**

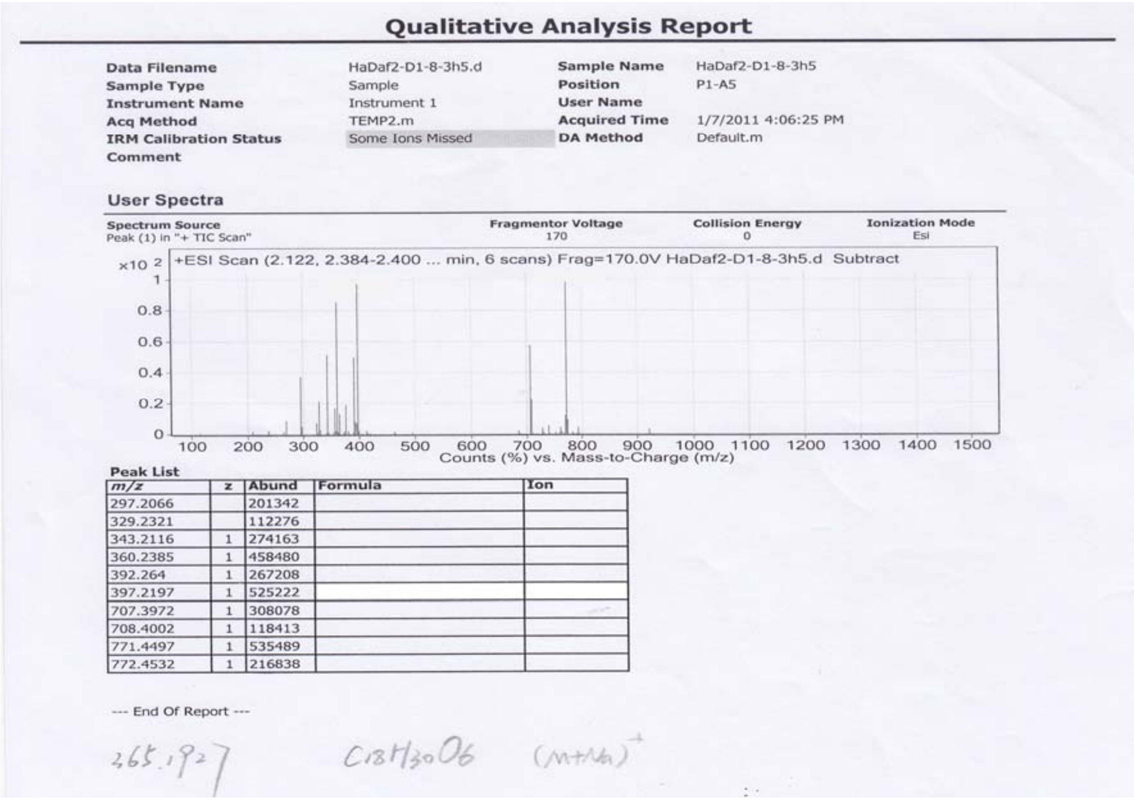

Qualitative Analysis Report

**Data Filename**	HaDaf2-D1-8-3h5.d	**Sample Name**	HaDaf2-D1-8-3h5
**Sample Type**	Sample	**Position**	P1-A5
**Instrument Name**	Instrument 1	**User Name**	
**Acq Method**	TEMP2.m	**Acquired Time**	1/7/2011 4:06:25 PM
**IRM Calibration Status**	Some Ions Missed	**DA Method**	Default.m
**Comment**			

User Spectra

Spectrum Source	Fragmentor Voltage	Collision Energy	Ionization Mode
Peak (1) in "+ TIC Scan"	170	0	Esi

+ESI Scan (2.122, 2.384-2.400 ... min, 6 scans) Frag=170.0V HaDaf2-D1-8-3h5.d Subtract

Counts (%) vs. Mass-to-Charge (m/z)

Peak List

m/z	z	Abund	Formula	Ion
297.2066		201342		
329.2321		112276		
343.2116	1	274163		
360.2385	1	458480		
392.264	1	267208		
397.2197	1	525222		
707.3972	1	308078		
708.4002	1	118413		
771.4497	1	535489		
772.4532	1	216838		

--- End Of Report ---

265.1927 $C_{18}H_{30}O_6$ $(M+Na)^+$

Figure S9. The ^{1}H-NMR spectrum of compound **3** (methanol-d_4, 500 MHz).

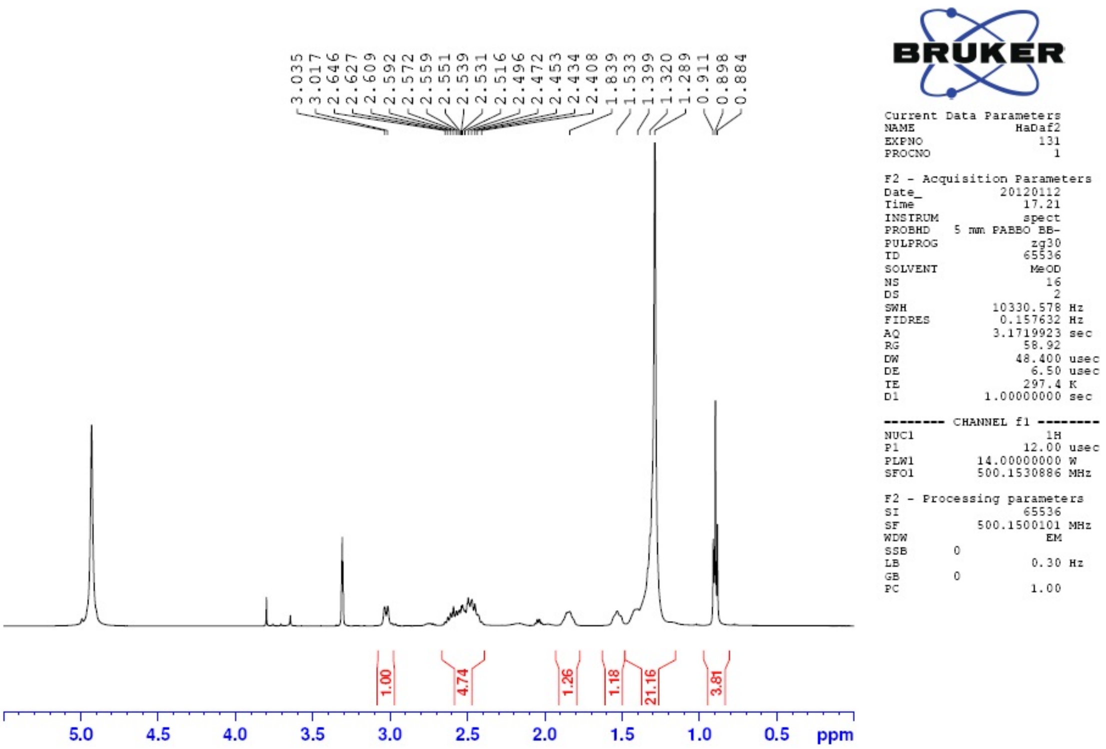

Figure S10. The ^{13}C-NMR spectrum of compound **3** (methanol-d_4, 125 MHz).

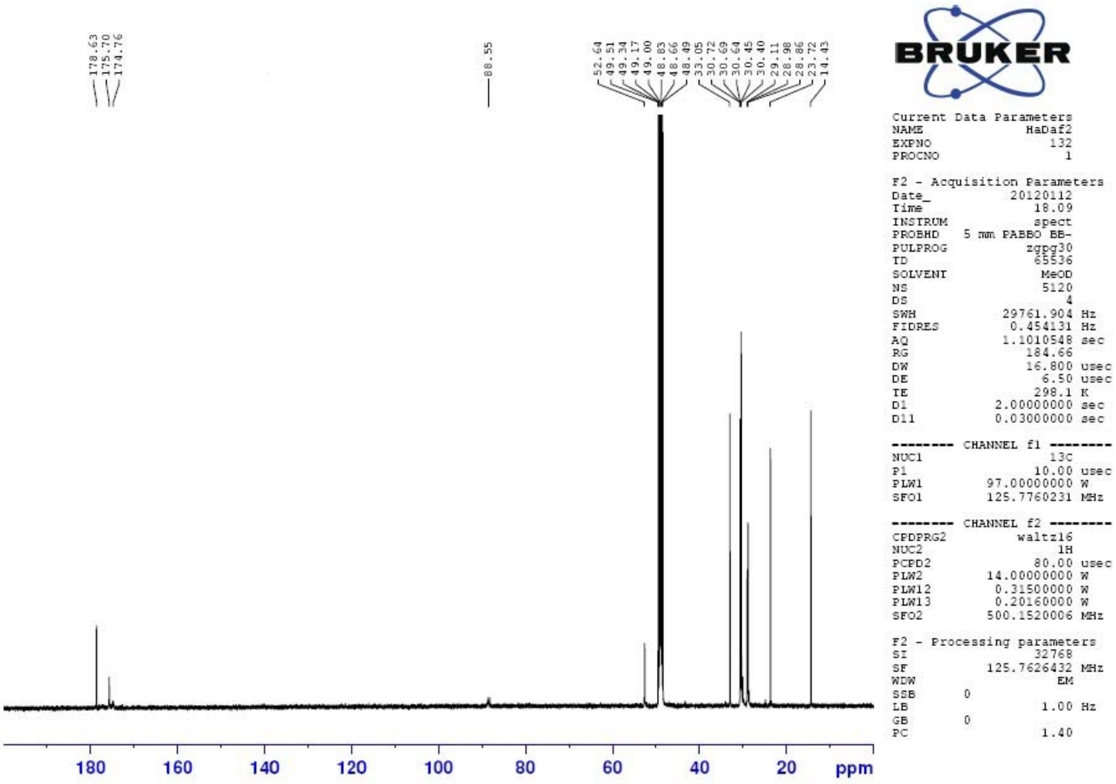

Figure S11. The DEPT135 NMR spectrum of compound **3** (methanol-d_4, 125 MHz).

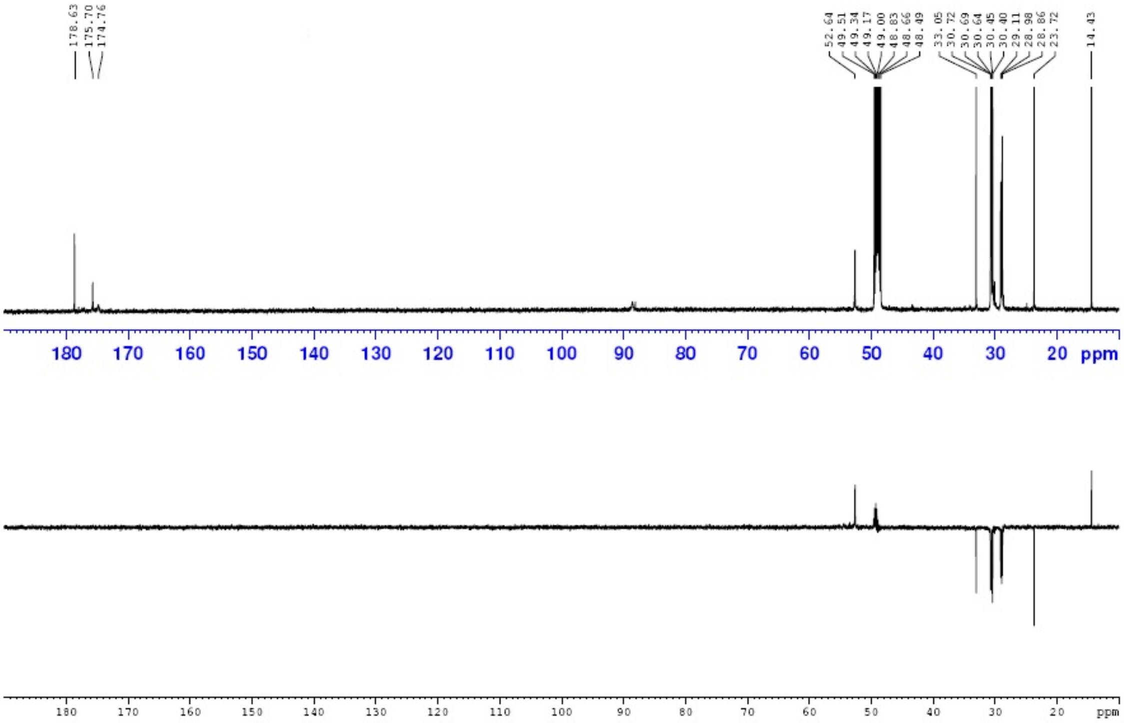

Figure S12. The HRESIMS spectrum of compound **3.**

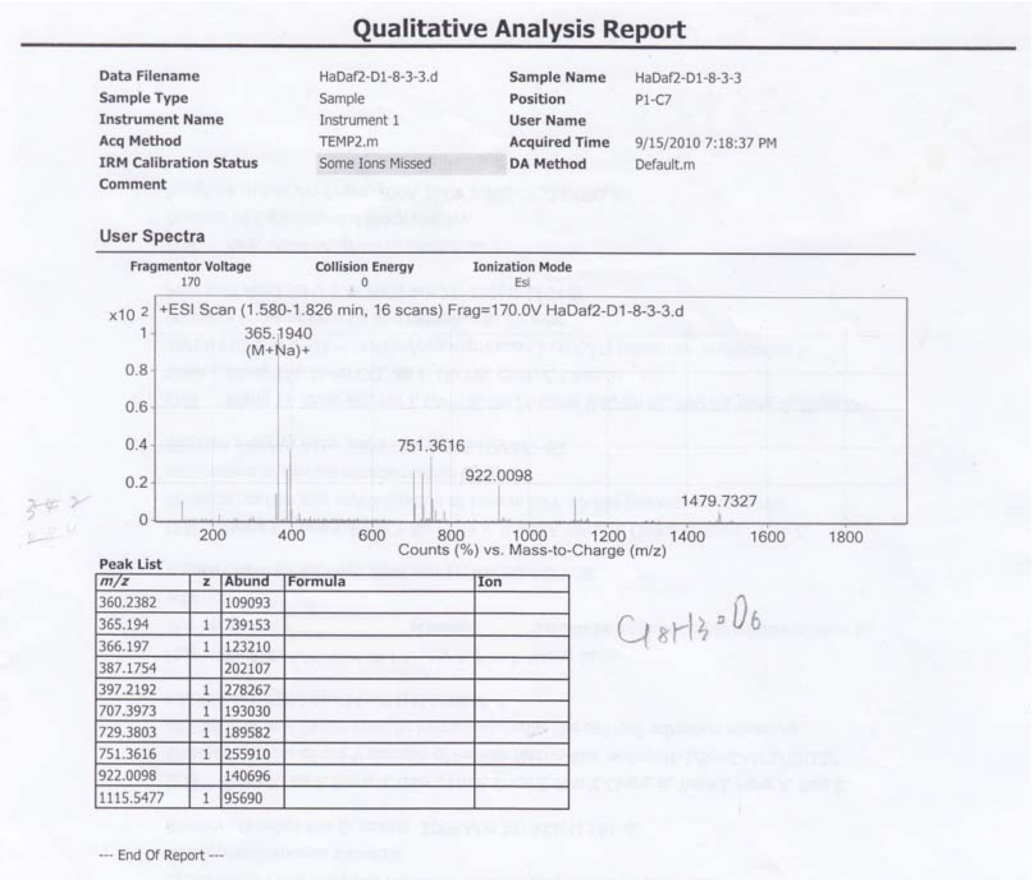

Qualitative Analysis Report

Data Filename HaDaf2-D1-8-3-3.d
Sample Type Sample
Instrument Name Instrument 1
Acq Method TEMP2.m
IRM Calibration Status Some Ions Missed
Comment
Sample Name HaDaf2-D1-8-3-3
Position P1-C7
User Name
Acquired Time 9/15/2010 7:18:37 PM
DA Method Default.m

User Spectra

Fragmentor Voltage 170
Collision Energy 0
Ionization Mode Esi

Peak List

m/z	z	Abund	Formula	Ion
360.2382		109093		
365.194	1	739153		
366.197	1	123210		
387.1754		202107		
397.2192	1	278267		
707.3973	1	193030		
729.3803	1	189582		
751.3616	1	255910		
922.0098		140696		
1115.5477	1	95690		

--- End Of Report ---
